# Affective Features Underlying Depression in Addiction: Understanding What It Feels Like

**DOI:** 10.3389/fpsyg.2019.02318

**Published:** 2019-10-17

**Authors:** Daniela Flores Mosri

**Affiliations:** Department of Psychology, Psychoanalytic Psychotherapy, Neuropsychoanalysis, Universidad Intercontinental, Mexico City, Mexico

**Keywords:** addiction, depression, affect, defense, subjective pain

## Abstract

Addiction poses a complex challenge in spite of all the progress made toward understanding and treating it. A multidisciplinary approach is needed and this paper attempts to integrate relevant neurobiological, behavioral, and subjective data under a common denominator described as a latent type of depression. It is called latent because it remains a silent syndrome due to two main reasons. The first one relates to the natural use of defenses against a predominant effect of chronic subjective pain, which arises from an ambivalent type of separation distress that compromises opioid regulation (PANIC system). Furthermore, it provokes a neurochemical cascade that impacts several neuromodulatory systems. The second reason is that such chronic subjective pain usually exhausts the natural defensive system, frequently leading the person to look for other resources such as the neurochemical manipulation of psychic pain. Thus, both the use of defenses and of psychotoxic drugs make the underlying depression hard to assess, even for the very person suffering from it. The causes, course and treatment of this type of affective configuration are discussed in this paper as an attempt to explain some of the difficulties so far encountered and to contribute to potential alternative lines of treatment.

## Introduction

There are many valuable approaches to understand addiction. Different perspectives explain its psychiatric, social, medical, historical, and psychological aspects. Addiction is about a behavior that goes beyond control. Neurobiological models explain craving, tolerance, withdrawal syndromes, kindling and its chronic relapsing vulnerability. Yet, seldom do they take into account the subjective states associated with the different stages of addiction. Addiction feels like something; affect should be used as a bridge concept to attempt an integration of some of the findings coming from different perspectives. Affect is felt subjectively and it can also be studied from a neurobiological point of view. It provides meanings that guide behaviors and thoughts. Emotions favor survival (Panksepp, 1998) and their regulation is compromised in addiction. This paper suggests the hypothesis that depression constitutes a key emotional configuration that can contribute to the initial voluntary decision of a person to use drugs. Some of those depressions may be apparently asymptomatic and thus remain undiagnosed. In general terms, if they do not meet clinical criteria (e.g., DSM or ICD), they do not exist. A psychodynamic exploration of a person’s emotional life can contribute to an early detection of problems that can later become clinical syndromes. The earlier they can be worked with, the fewer risks for the person. From a psychoanalytic point of view, these depressions can be called “latent.” If they are felt, there are neurobiological correlates that should be used to better understand them.

This latent type of depression is proposed to exist prior to addiction and to contribute to its etiology. It relates to early experiences of ambivalence with the primary caretaker that lead to chronic separation distress. This paper will not use clinical materials to illustrate its hypotheses; the reader can find examples in the literature (e.g., Fine, 1972; Gustafson, 1976; Johnson, 2009, 2010; Flores Mosri, 2017a). Only a few examples will be used to illustrate some of the hypotheses and theory described throughout the paper. I suggest that ambivalent affective experiences with the primary caregiving object may result in neurochemical and subjective dysregulations that could contribute to help explain the use of psychotoxic drugs and its implicated behaviors. Such dysregulations work in cascades in which one dysregulation leads to another connection within a spiral loop that relates to depressive feelings. Some alternative aspects for treatment will be briefly discussed.

## The Impelling Need of an Integrative Approach and Its Caveats

Addiction is a complex topic. It has been studied from different perspectives resulting in a plethora of knowledge that has been helpful to design different lines of treatment. Yet, it still constitutes a major health problem. It is a disease that poses challenges and questions that can hardly be answered from one perspective. Addictive disorders can be studied from behavioral, psychiatric, social, anthropological, and neurobiological perspectives amongst others. If such an endeavor is done separately, the result is that the topic of study is split and thus only partially understood. An integrative approach attempts to bring together as many findings as possible. This challenge comes with enormous methodological difficulties. Epistemologically, the different viewpoints may seem separate and impossible to bring together. As much as that may seem reasonable, the loss in such a position is to give up on potential dialogs between disciplines. If addiction was only a behavioral, neurobiological, or psychiatric disease, then it would be appropriate to stay faithful to each of the discipline’s methods. This paper attempts a dialectical perspective by highlighting the most commonly neglected aspect of addiction, which is its subjective experience. Its neglect does not seem casual. It is about the most difficult part to study, precisely because subjectivity is not an object, but an experience, referred to by Solms and Turnbull (2002, 2011) as the first-person perspective that should accompany third-person perspectives when it comes to understanding the mind. Describing subjective aspects can be difficult and not generalizable, but patients suffering from addictive disorders are people who experience and feel through the whole process, ranging from the premorbid stages to the morbid and deadly ones. Partial understandings may contribute to more people losing their lives. Studying addiction separately strengthens scientific coherence, but if that knowledge is not brought together at some point, it also flaws its comprehension and suggested treatments.

Hence, this paper attempts to present a potential dialog between disciplines that study addiction. It is a disease that affects behavior, brain, body, and mind. All of these components have an effect on one another. I take a dual-aspect monist position (Solms and Turnbull, 2002, 2011; Panksepp and Solms, 2011). The mind needs the brain to exist. Panksepp (2011a) proposed the term “BrainMind” or “MindBrain” to emphasize that mental processes or internal experiences are linked to neural dynamics. Such a position allows for improved understanding of behaviors and their motivations from a monistic perspective, that acknowledges that the brain is a feeling organ and the seat of the mind. Studying addiction is a good example of the latter. It is seen in behaviors that relate to neurochemical circuits whose modifications produce feelings. Panksepp (1998) devoted his life to the study of affective neuroscience. He claimed that all mammalian organisms share subcortical basic emotion circuits that guide instinctual behaviors. He distinguished systems and capitalized their names to highlight that words that can be used in a colloquial way, in the context of affective neuroscience, mean the activation of a specific neural network that relates to affective feelings and specific behaviors. He named those seven circuits, the SEEKING, PANIC/GRIEF, RAGE, FEAR, LUST, CARE, and PLAY systems. These words are used in that same way throughout this paper and I will explain their features in more detail when necessary for the purposes of this manuscript. All of them aim at enhancing the chance to survive. For a thorough description of each circuit, the reader is referred to Panksepp’s (1998) book *Affective Neuroscience*. Since this paper is about the subjective experience of addicted people, the correct understanding of these neurobiological circuits is crucial.

This paper focuses on three different categories to study addiction: the behavioral, the neurobiological, and the subjective. The challenge is to try to integrate indicators that belong to different levels of analysis. This paper represents only an attempt to propose hypotheses that respond to the many people suffering and dying from addictive disorders. We can no longer ignore the neurobiological and subjective aspects that display in their pathological behaviors. They all relate to one another and the least we can do is to try to suggest some hypotheses to be further researched.

### The Stages of Addiction and the Importance of Subjectivity

**FIGURE 1:**
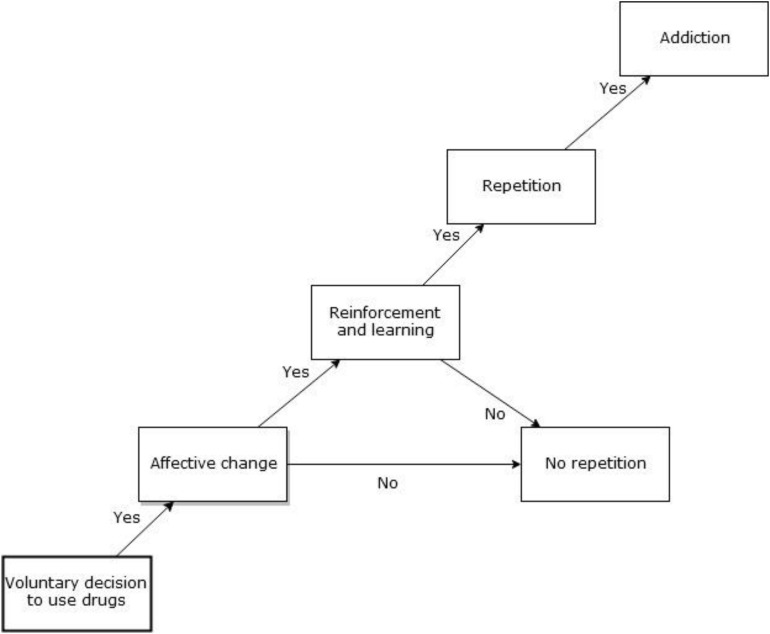
From initial drug use to addiction. The figure describes how an initial voluntary decision to use drugs can lead to addiction, particularly when an affective change is reinforced and learned.

Addictive behaviors usually start with the voluntary decision to use a drug (Panksepp et al., 2002; Volkow and Morales, 2015). For the drug to be reinforcing, it must change a subjective state quickly, so that a direct association is established between the consumption of the drug and mood modifications. An emotional memory will be formed and reinforced as long as the effects of the drug either produce a positive feeling or reduce a negative feeling. Adding genetic, developmental and environmental vulnerabilities, a person who has tried drugs may or may not develop an addictive disorder (see Figure 1). Drugs of abuse increase the release of mesolimbic dopamine involving the VTA and the NAcc pathway. This initial phase of trying the effects of a psychotoxic drug may be followed by repeated and frequent use that may eventually lead to a gradual urge to use the drug, which slowly results in an involuntary decision guided by regulatory brain modifications. Despite other factors being present, the frequent repetition of the consumption of a drug of abuse can be enough to modify the VTA-NAcc pathway and to produce a chronic acquired brain disease (Volkow et al., 2016). At this stage, an addictive disorder can be diagnosed, characterized by a psychological and/or neurobiological dependence. Addicted people will prioritize the drug consumption over other rewarding behaviors, stimulating the mesolimbic dopamine pathway even to the point of death (Olds, 1977; Panksepp et al., 2002).

According to Volkow et al. (2016), three stages of addiction can be distinguished: (1) binge and intoxication, (2) withdrawal and negative affect, and (3) preoccupation and anticipation. The stage of binge and intoxication is characterized by an increase in dopaminergic activity; all addictive drugs increase the release of dopamine which has been interpreted as a reward signal linked to associative learning. Dopamine is released to anticipate a response which eventually strengthens synaptic connections, leading to LTP and LTD, involving glutamatergic activity (Wright and Panksepp, 2012). The course then goes from experimentation with drugs to addiction, which implies progressive neuroadaptations in the brain, i.e., an acquired disease of the brain (Volkow and Koob, 2015). Conditioning leads to sensitized learning and memory formation recruiting the VTA and the NAcc, which establishes habits and routines along with the dorsal striatum. Other key structures that regulate dopaminergic activity include the amygdala and the hippocampus (Wright and Panksepp, 2012). The mesolimbic dopamine pathway is modified by the repeated use of drugs resulting in craving, which will motivate the patient to look for the drug and to use it. The brain is gradually changing and getting ill. Addiction weakens brain regions involved in executive functions, such as decision making, inhibitory control, and self-regulation. This prefrontal function impairment contributes to repeated relapse. The patient’s will is compromised (Johnson, 2013) and there is loss of self-control. From the subjective perspective, the users experiencing those modifications do their best to try to explain these new feelings to themselves. They initially try to deny the loss of self-control. They frequently state that they can quit using drugs whenever they want to. But they also clarify that they do not want to stop. This type of sentence is a clinical indicator that the patient has lost control and that the dopamine mesolimbic system may have suffered neuroadaptive modifications. As seen in Olds (1977) findings, the users’ predominant goal becomes to stimulate this pathway. Negative consequences of drug abuse will be ignored and previous interests will be left behind. Dopamine release in addictive disorders starts to feel bad when it gives the experience of a positive expectancy of satisfaction that never actually comes (Panksepp et al., 2002). The prediction never meets real sensory input and satisfaction (Schultz, 2006, 2016). Dopaminergic neurons keep firing due to the effects of drugs. This constitutes a pathological activity that means that the drug in itself is not rewarding.

Hence, the constant firing of dopamine does not mean pleasure and object-finding; it means expectation of finding satisfaction. Dopamine release then turns into a frustrating experience, yet users continue their neurochemical stimulation. From Panksepp’s view of a SEEKING system, only the actual finding and consumption of the satisfying object stops dopaminergic release in the mesolimbic dopamine pathway (Panksepp, 1998; Schultz, 2002), meaning that dopamine firing will only stop when the object is being consumed. Then a different pathway is activated, a “liking” system (Berridge et al., 2009) that is different from a “wanting” dopaminergic system. The satisfaction is related to the activity of several neurochemicals, including increases in opioid activity (Panksepp et al., 2002; Burgdorf and Panksepp, 2006). The illusion experienced in addiction means that as long as dopaminergic neurons keep firing, the mu and delta opioid receptor activity related to satisfaction is not active. Thus, addicted patients only experience the expectation of a positive feeling, but not the pleasure of actually finding a satisfying object. Addiction is a frustrating and failed illusion. The more frustration the user experiences, the less dopaminergic activity their brain shows. Neurobiologically, the dopamine system is downregulated resulting in feelings of hopelessness (Watt and Panksepp, 2009). It now has neuroadaptations that compromise its capability to fire in search of a motivated exploration of its environment.

**FIGURE 2:**
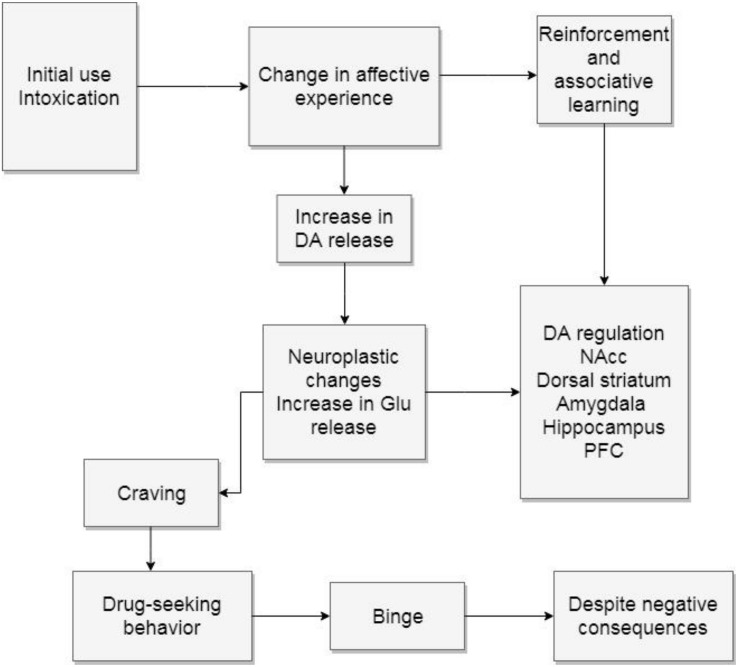
Binge and intoxication. The diagram describes how initial drug use can cause neuroplastic changes. DA, dopamine; Glu, glutamate; NAcc, nucleus accumbens; PFC, prefrontal cortex.

To sum up, the first stage of addiction involves the experience of intoxication, which, if repeated, will in turn lead to a decrease in the ability to feel motivation and pleasure. The neuroplastic changes imply an increased release of glutamate that impacts the NAcc, the dorsal striatum, the amygdala, the hippocampus, and the PFC. All these structures regulate dopamine firing. Because the dopamine pathway has been modified, the user’s motivational feelings and behaviors will be compromised (see Figure 2).

The second stage for Volkow is withdrawal and negative affect. This model states that regular rewards lose their former motivational power, due to the downregulation of the dopamine mesolimbic pathway. At the same time, there is also a hyperactive impact on the extended amygdala circuitry that produces negative affects related to withdrawal. Users will try to avoid these negative feelings, constituting a new type of negative reinforcements. They now have a powerful reason to repeat drug use, which is to alleviate from withdrawal symptoms. Anxiety and stress are predominant feelings during this stage and they can in turn lead to irritability and aggression. Several alterations in the regulation of the HPA are observed (Volkow et al., 2016), enhancing the release of CRF. Volkow and Morales (2015) have called the allostatic changes that lead to the use of drugs to try and alleviate withdrawal symptoms, the “dark side of addiction.” They implicate the amygdala, the BNST and the NAcc shell. There is also an upregulation of dynorphins linked to the dysphoric feelings that characterize this stage. Furthermore, neurochemicals related to positive emotions, such as enkephalins and endocannabinoids, are downregulated. The lateral habenula is also impacted by the use of drugs since it is another regulator of dopamine firing. It is active when positive expectations fail to happen, as well as in the presence of aversive stimuli.

**FIGURE 3:**
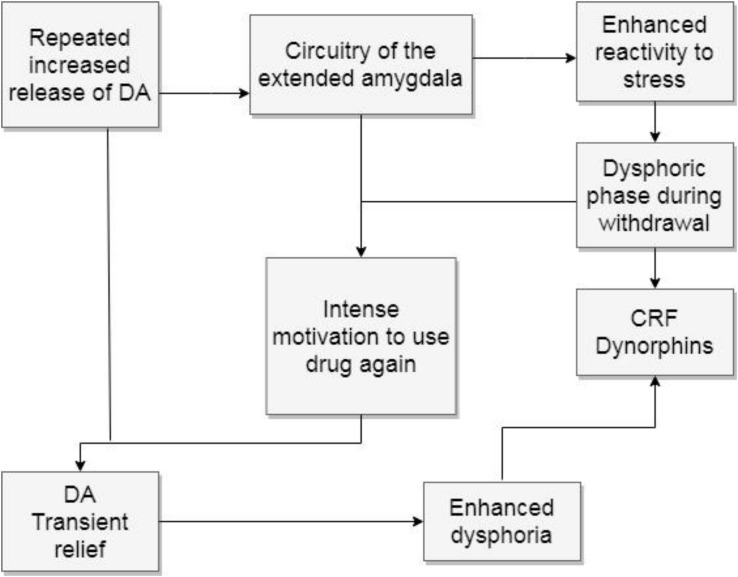
Withdrawal and negative affect. The figure shows how the repeated increase of dopamine release causes a hyperreactivity to stress by recruiting the circuitry of the extended amygdala. The attempt to escape dysphoric feelings increases the motivation to use drugs again, deepening dysphoric feelings. DA, dopamine; CRF, corticotropic releasing factor.

To summarize, this stage recruits what has also been called the “antireward” system (Volkow and Morales, 2015). It implies an enhanced reactivity to stress which yields negative emotions when the drug is withdrawn. These dysphoric feelings result in an intense motivation to escape the discomfort, which the drug can help mitigate by a renewed increase of dopamine release. Yet, since the dopamine release gradually diminishes, the relieving feelings are also gradually less effective, leading users to increase doses and frequencies of drug consumption. They binge, which in turn deepens the dysphoria during withdrawal. Users are more prone to overdose at this stage (see Figure 3).

**FIGURE 4:**
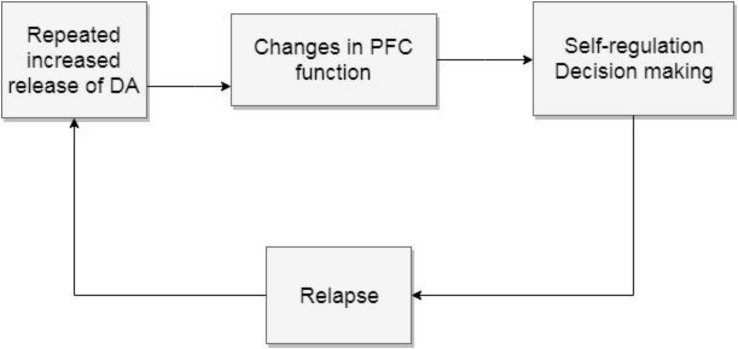
Preoccupation and anticipation. The repeated increase of mesolimbic dopamine release changes the PFC functions, particularly self-regulation which leads to relapse to start the cycle again. DA, dopamine; PFC, prefrontal cortex.

Withdrawal symptoms worsen previous stressful feelings, favoring some of the features of the third stage proposed by Volkow, preoccupation and anticipation. This stage emphasizes the compromise of the PFC, which impairs self-regulation and other executive functions. The PFC inhibits and regulates behavior (Anderson et al., 2016). In addiction the user can no longer make the decision to stop. This known progress of addictive disorders enhances the importance of detecting negative affects that can lead to the initial voluntary decision of using drugs, since this stage means that treating users will represent a complex challenge. Functions such as attribution of salience, decision making, planning, and monitoring of actions are modified. The top-down regulation of emotional circuits is compromised leading to an inability to resist the urge to use drugs (see Figure 4).

### Addiction Feels Like Something: The Contributions of Ambivalent Separation Distress and Latent Depression

As seen before, the various stages of addiction add up and result in a compulsive cycle that is difficult to interrupt. Users face complex consequences of drug use and are somehow expected to reflect on the negative outcome and be able to stop. Giving information to prevent the use of drugs is useful and responsible, but not enough. The latter can partially be explained by acknowledging that it is not an illness related to thinking and stopping. Addiction is an illness related to emotional states. Users try to change the way they feel through using exogenous agents that have an impact on their neurochemical emotional pathways, hence the importance of affective neuroscience. What starts and reinforces addictive behaviors is the subjective experience associated to the comparison of an initial affective state against the modified state derived from using a drug. Thus, what happens in both states should be studied which is the topic in this section.

Addiction is a multi-caused illness that cannot be prevented or solved with simple actions. Wurmser (1974) applied Freud’s idea of complementary series to highlight that addiction is caused by several factors. He suggested the existence of a precondition, i.e., a narcissistic injury, that is present before the onset of compulsive drug use, but that is not sufficient to explain it. Next, there is a specific cause, which is present in all cases but not enough to cause the disorder, unless the precondition is there too. Wurmser saw the specific cause in an emotional reason derived from a narcissistic conflict, whose related affects are anxiety, depression, disillusionment, and rage. Since they are all negative affects, there is a need to escape from experiencing them. The third set of reasons can be referred to as concurrent, which operate alongside the specific causes and preconditions, but they are not sufficient on their own to originate the syndrome and they are not present in every case. Examples of these factors may be seen in socio-cultural uses of drugs, philosophical questions and protest. The fourth type is the precipitating cause. It refers to the factor preceding the beginning of the disease, e.g., the availability of the drug.

This hierarchy of causes indicates that the vulnerability to addiction depends on a combination of components that explains why some users try drugs and do not become addicted, and why some others will. Research shows that genetic factors also play a role in the vulnerability to addiction (Kreek et al., 2005; Bierut, 2011), but cannot explain the initiation of drug use alone. An epigenetic perspective seems more appropriate and recruits other components.

To understand addiction, it is important to consider that behaviors have different sources of motivation. Subjective affective experiences guide actions. In Panksepp’s view, there is a basic SEEKING system that explores the environment in search of resources to survive. But SEEKING does not have an object. Other systems inform it of what it should look for, e.g., water when thirsty, food when hungry, a caretaker when lonely and helpless. Thus, reinforcements and conditionings are mediated by an affective experience that helps organisms to learn from experience and use memory to build expectations. The SEEKING system depends on specific neurochemicals and neuroanatomy. This is why it is important to study behaviors, such as addiction; its motivational and neurobiological aspects should also be taken into account.

In terms of using drugs, I hypothesize that depressive feelings contribute to initial consumptions. If the behavior is repeated, then these initial depressive feelings are enhanced with a neurochemically induced addictive cascade. To explain this proposal, I will describe two phases. The first one characterizes a latent type of depression that would constitute a precondition of addiction, meaning that it has its origins in developmental factors that will constitute in a *pre-addictive* phase. The second phase derives from repeated drug use by causing a deep neurochemical dysregulation that takes the form of anxiety and depressive feelings in an *addictive* phase. The initial depressive feelings merge with those provoked by drug use, intensifying the negative affective experience that users describe.

As important caveats, the reader is reminded that individual drugs produce different neurochemical and subjective effects (e.g., stimulant, depressant, psychedelic, or pleasurable effects). Still, drugs of abuse share common mechanisms that explain addiction in general (Robinson and Berridge, 1993). The differences between types of drugs are beyond the scope of this manuscript. Another important reminder is that the following contents constitute only hypotheses that require further research. They derive from available data in the literature and are complemented by clinical observations that can be found in any case study previously published. The main hypothesis is that, additional to the various factors that contribute to the onset of addiction, depression plays an important, but not sole, role in the causation of addiction.

#### Pre-addictive Phase: Depression as a Precondition of Addiction

When individuals make the voluntary decision to try drugs, they frequently relate their decision to curiosity or recreational purposes. Some other intentions can be relevant and inaccessible to conscious awareness. Not all users become addicted, but they are all modifying affective states through the use of psychotoxic drugs. At this stage, repeated use means that the person wants to modify an emotional state again, despite the awareness that it damages the body and that it can result in both psychological and physiological dependence. To explain this paradoxical behavior, Freud (1898) suggested that addiction could be interpreted as compulsive masturbation, meaning that users were in a compelling search of pleasure. His hypothesis makes sense when users express that they want to use a drug for recreational purposes. The question is, if a person feels good, why would they need to feel better? On the other hand, there are hypotheses that state that drug users are trying to alleviate from negative emotions (Loose, 1998). As said before, behaviors cannot be understood without considering their motivations. In either case, the principle that underlies both hypotheses is that the user is aware that feelings can be modified by using drugs. An associative learning is established. Emotional states depend on neurochemical features that can be changed by introducing exogenous molecules into the body. Users become “wild” psychiatrists. The principles they are using correspond to those of psychopharmacology.

Thus, some users will find it hard to stop using drugs whenever they need to modify their emotions. As discussed before, repeated use will eventually lead to neuroplastic changes that contribute to a chronic brain disease (Volkow and Morales, 2015). Once the brain is modified, it needs the drug, becoming a slave of its own defense, i.e., while trying to feel better, it becomes ill. The latter constitutes the addictive paradox.

Users of psychotoxic drugs usually share an initial negative affective state and they try to self-medicate (Khantzian, 1985, 2003) with or without conscious awareness of it. Negative feelings indicate that action is required to solve a problem or an unmet need (Panksepp, 1998; Solms, 2018, 2019). If a solution is not available, the negative feelings persist and defenses are then used to relieve the unpleasant feelings at least partially. Yet, the unpleasant affect is there to drive the person into some sort of action that can look for a satisfying object (Damasio, 2010; Panksepp, 2010; Solms, 2019). No defense will be able to fully relieve the person from their negative feelings until a specific action (Freud, 1895/1950) is taken to meet a homeostatic need. Defenses will expire if the person does not find a way to finish with the tension that arises from unmet needs. Suffering may become a chronic, negative emotional state characterized by frustration, which may in turn cause anger, anxiety, stress and hopelessness. This affective configuration summarizes depression. Defenses, particularly manic ones, render the depressive affect into a discreet but constant feeling. This is why this type of depression is called latent.

Metapsychological analyses state that depression starts with the experience of loss (Freud, 1915/1917). The normal process of mourning becomes pathological when there is a narcissistic type of object which is characterized by ambivalent feelings toward the lost object. There is guilt related to aggressive feelings toward the object that explain self-aggressive behaviors and that isolate the person from the outside world. Freud suggested that suicide can be interpreted as a wish to kill the object.

The narcissistic aspect of depression has been studied by several psychoanalytic theories. Balint (1968) related it to the concept of a basic fault that expressed the discrepancy between what a child needs and what the environment can provide. Marty (1990) also spoke of important failures in early maternal care as the origins of essential depression. This type of experience favors the splitting mechanisms of object representation described by Klein (1946), Fairbairn (1954), and Kernberg (1975). Bergeret (1974) described an anaclitic type of object relation in which a contradictory dependence is experienced, particularly in borderline states. Anaclitism implies the need for an object that is rejected as soon as it is near. When the object is away, it is desired back only to be rejected again. Marty (1958) called this type of object relation allergic, expressing that same paradox. Bergeret (1974, 1975) suggested that the origins of this type of anaclitism come from a narcissistic disease in which parents show emphatic ambivalent feelings for their children. Parents promise to love their children only under the condition of staying close to them. In this model, the children are not loved unconditionally; parents make them feel that they have not been loved because they have not been good enough, which in turn leads to ego ideal pathology. Children feel as though they had lost their parents’ love due to not being good enough. I have suggested a paradoxical mourning process for this context (Flores Mosri, 2017a, b) in which children are trying to mourn for a lost object that they never actually had, thus, it could not be lost. An impossible mourning process guarantees a melancholic process that becomes chronically painful.

Ambivalent feelings from parents to children are also described by Kalina (1997, 2000) who states that addicted individuals are raised in environments of contradictory messages given by the parents, who tend to be narcissistic and depressive. These circumstances lead to inconsistent feelings that compromise an experience of basic trust (Erikson, 1950). The latter psychodynamic proposals come basically from clinical observations by many authors (e.g., Ferenczi, 1949). Psychoanalytic descriptions may result exhausting or too abstract to be taken into account in other clinical or research contexts. Yet, their accounts are important because they examine subjective experience in depth. A change from the exploration of unconscious fantasies to the effects of real experiences in psychoanalysis was emphasized in Bowlby’s (1988) work. He highlighted the importance of an early and consistent attachment relationship upon which a child’s future adapting mechanisms depend. These facts take us back to the PANIC/GRIEF system proposed by Panksepp (1998).

From an affective neuroscience perspective, the condition of helplessness of young mammalian organisms is the key to having a PANIC/GRIEF system (Panksepp and Biven, 2012), which relates to Bowlby’s attachment system. Babies need to be taken care of or they die. Survival entails SEEKING for homeostasis through a primary caretaking figure. The mother is the person best suited for the role, as hormones and a CARE system are the perfect match to a baby’s PANIC needs. Separation feels bad because it leaves the helpless baby exposed to various dangers that make them call for a reunion with the primary caretaker. The separation distress circuit runs from the dorsal PAG to the ACC. PANIC is felt as a subjective form of pain derived from a need for the object. The child’s instinct makes them feel lonely and sad, thus they protest by using separation calls that intend to get the caretaker’s attention. If the caring person is able to reunite with their child, the PANIC activation stops and with it the psychic pain that was suffered. If such an ideal context does not happen, PANIC activations will occur more often or even chronically. This can be the circumstances of ambivalent and inconsistent caretakers.

If affect is ambivalent and obscure, there is little chance of succeeding at problem resolution in the future. Instincts favor survival (Panksepp, 2011a) and children will try numerous times to meet their needs. If they receive little or faulty assistance from their primary caretaking figures, not only can they not learn, but they will also experience PANIC activations. They feel insecure, frustrated and lonely, which will eventually lead to sadness and probably to despair. All of these feelings entail KOR activation and dynorphin activity. This in turn diminishes dopamine activity in the SEEKING system, which is behaviorally seen as a lack of motivation and energy to look for a way to solve problems. This first cascade effect is enough to constitute a depressive affect (Watt and Panksepp, 2009; Panksepp, 2010; Panksepp and Watt, 2011). Separation distress may become a persistent low activation and feeling of PANIC/GRIEF. Looking for a caretaker may also become chronic and may downregulate other social instincts (Panksepp et al., 2014) such as those involved with having fun with others (PLAY), of having a love partner (LUST) and even taking care of others (CARE). Protracted unsatisfied needs also cause hyper-reactivity of the HPA axis and the amygdala, which entails an increased release of CRF and various corticosteroids (LeDoux, 1996; Watt, 2017). Anxiety and stress highly contribute to the search of psychotoxic agents (Volkow et al., 2017). Because of the massive and diverse array of negative feelings it recruits, depression constitutes a part of the vulnerability toward addiction.

Relating the findings of affective neuroscience in terms of emotional experience, it can be summarized that low activity of dopamine in the mesolimbic pathway results in a depressive feeling characterized by apathy and hopelessness; the expectation that a chronic state of separation distress will ever be solved diminishes. Yet, people who experience ambivalent attachment feel separation distress and call for a caring object that they cannot rely on. This leads to psychoanalytic descriptions of anger in depressed people (Abraham, 1911; Freud, 1915/1917; Wurmser, 1974; Dodes, 1990). As explained by Freud, in depression the object is introjected and aggression is turned against it, hence it is addressed against the self. Some addicted patients have expressed in abstinence that they needed their parents to know that they had not been good enough. It is basically anger against the narcissistic rejection that makes people feel unworthy (Freud, 1914).

Thus, two indicators of depression can be extracted from psychoanalytic contributions. The first one is a depressive affect experienced as hopelessness, sadness, and guilt. The second one is related to self-aggressive behaviors. These indicators should be searched for in future predictive studies of the vulnerability to addiction. The negative effects can hide behind the use of multiple defense mechanisms, particularly manic defenses. Thus, a proper psychodynamic exploration may be needed to distinguish them. In terms of self-aggressive behaviors, they manifest and are reliable indicators: people are willing to engage in self-damaging conducts. A higher risk of using drugs can be predicted in these people.

In sum, when depression is acknowledged, it is possible to understand why these patients feel curious to try drugs. Addicted patients frequently find themselves with a lack of resources to solve their problems. They developed inefficient templates to attempt to meet their needs and these patterns do not seem to learn from experience. These templates represent major prediction errors (Friston, 2010; Solms and Friston, 2018) since they keep failing, and cannot take into account prior and posterior evidence to modify the way to interact in the world. A negative affective subjective experience is then predominant.

#### Addictive Phase: The Subjective Experience of the Stages of Addiction

Drug users do not want to feel bad and the addictive process ensures that they will feel progressively worse. Some psychodynamically oriented clinicians may think of the repetition compulsion and the death drive because there is little opposition from users to engage in self-aggressive and self-damaging behaviors, including addiction. Some of them are not aware that there are important brain modifications that explain much of what is seen in addictive disorders. The latter guarantees that wrong treatment strategies will be used.

Addictive drugs are tempting and seductive. They help the subjective sensation, but they certainly solve no need. After the intoxication stage finishes, the negative feelings are back, usually adding a new source of frustration related to the loss of a better affective state, additional to withdrawal symptoms. From a psychological perspective, the user is motivated to repeat the search of drugs in order to achieve psychotoxic relieving and/or pleasurable effects. Repetitive drug use eventually leads to brain modifications (Volkow et al., 2016) that can hypersensitize (Robinson and Berridge, 1993; Berridge et al., 2009) an incentive and motivational system (“wanting” system), also known as the “reward” system after the self-stimulation observations by Olds and Milner (1954), and renamed and enhanced in Panksepp’s basic emotion systems as a SEEKING system (Panksepp, 1998; Panksepp and Biven, 2012). As explained before, dopamine is a key neuromodulator of this mesolimbic pathway (Panksepp et al., 2002; Panksepp, 2011a).

Multiple interpretations regarding the role of this circuit have been made and it is important to review them to try to understand the subjective feeling of using a drug during the morbid phase of addiction. The predominant view states that the mesolimbic dopamine pathway is “the reward pathway” and is thus involved in addictive behaviors (Volkow and Morales, 2015). From this perspective, the hypothesis that drug users look for pleasure makes sense, since they would be looking for a reward. However, recent findings suggest that this pathway is not related to rewards, but to the search for rewards (Panksepp, 1998; Schultz, 2002; Volkow and Morales, 2015). For Panksepp, it is one of the seven basic emotion systems that constitute mammalian instincts oriented toward survival. SEEKING produces emotions during its activation. It gives feelings of excitement and positive expectation (Wright and Panksepp, 2012). It can then be inferred that addicted people are looking for a positive feeling of motivation toward life. Psychostimulants, such as cocaine and amphetamines, directly activate the dopamine mesolimbic pathway. Users report that they feel excited about the plans they make during intoxication, that they feel hopeful.

Specifically addressing the separation distress feelings, the EOS also plays a part in addiction. Drugs such as stimulants, opiates, cannabis, and alcohol increase opioid activity in the NAcc and the VTA (referred in Volkow and Morales, 2015). These drugs can stimulate the “liking” system (Berridge et al., 2009), causing pleasure. However, their role in addiction may also be explained when it is understood that the subjective pain derived from separation distress involves dynorphin release (Panksepp and Biven, 2012), which in turn inhibits dopaminergic activity in the VTA and the NAcc. Withdrawal symptoms increase KOR activity, hence decreasing dopamine release, which in turn worsens the depressive symptoms previously described, along with an enhanced activity of CRF, which also plays an important role in depression (Watt and Panksepp, 2009). All of these components contribute to a depressive shutdown that users may try to decrease by using drugs that diminish the feeling of depending on others, as described in anaclitic relationships. Many drug users aim at social self-sufficiency. They would prefer not to need others because, in their experience, they are not reliable and they hurt. They would not like to risk an unpredictable outcome by relating to others. The use of drugs that have an effect on the EOS can be particularly efficient to achieve this goal; it brings a feeling of not needing anyone (Johnson and Faraone, 2013; Johnson and Flores Mosri, 2016). Opioids coincidentally have powerful antidepressant effects (Panksepp, 2015; Yovell et al., 2016).

Adding to the depressive cascade, Watt (2017) has suggested that CRF plays an important role in separation distress. Furthermore, it can be related to stressful experiences starting early in life due to diverse types of trauma, which in turn lead to permanent alterations of the HPA axis and thus, a chronic upregulation of CRF (Heim and Nemeroff, 1999). Watt and Panksepp (2009) stated that anything that restores the homeostatic regulation of the HPA axis has therapeutic effects on depressive symptoms. This constitutes another reason to hypothesize that addictive behaviors comprise an attempt to alleviate depression and also anxiety.

Therefore, as much as addicted people may seem to be in a slow process of committing suicide, they are also fighting against depression. They use drugs to alleviate from negative feelings derived from separation distress while they also try to experience motivation. Yet, as already described, drugs overstimulate dopamine mechanisms (Volkow and Morales, 2015; Schultz, 2016) impeding the search for other satisfying objects that could eventually lead to authentic rewards that enhance opportunities to adapt and feel well. Addicted patients are trying to live while they are killing themselves. They live in a paradox and harm their own body and, thus, may die while trying to survive. They dissociate their body and their mind (Kalina, 1997, 2000). They act as though they could survive in spite of the damage and deterioration of their organism.

In sum, the neurochemical features related to depression entail a complex cascade that interacts with the effects of drugs in the basic emotion systems. The “premorbid” conditions only worsen as a result and enhance depressive feelings. Addiction is an illegitimate resource to try to solve problems. It promises to stop depressive feelings, but not only does it not deliver, it will also make the initial situation worse. Addiction is a failed attempt at surviving. Addicted people usually refuse to ask for legitimate help and they find it hard to accept it when they can have it. Addiction has hijacked the mind.

### Treatment

Treating patients who suffer from addiction is a challenging endeavor. Most models are integrative and well planned but the nature of addiction itself poses all sorts of impediments. This is one of the reasons why there is an impelling need to complement existent models and to improve our understanding of addiction. As highlighted throughout this manuscript, its causes represent various dimensions that interact in complex ways, motivating clinicians and researchers to deepen our comprehension of its features.

Understanding the subjective experience of patients represents a chance to prevent and treat factors that may remain neglected. The hypothesis of a latent depression underlying addictive disorders entails the need for identifying features that may remain too “silent” to be taken into account. The need of metapsychological assessments is thus highlighted. Because of their own nature, latent or essential depressions (Marty, 1966, 1990) may seem asymptomatic syndromes and thus, tend to remain untreated. If they are better understood and diagnosed, more people may have the chance to be treated in terms of the clear vulnerability to addiction that it represents. People who experience a depressive cascade are more likely to try to regulate their neurochemical functioning because they feel bad. They will lose self-regulation and self-inhibition which will in turn make the addictive disease increasingly harder to treat.

Life implies that a perfect homeostatic state is never met, but is always searched for. Panksepp stated that affects are always conscious because they are felt (Panksepp and Biven, 2012) constituting a basic kind of subcortical affective consciousness (Panksepp, 2011b; Solms and Panksepp, 2012; Solms, 2013, 2019) that guides behavior at all times. SEEKING of satisfying objects is mediated by dopamine and it is the same pathway that addiction recruits. Hence, addiction is a symptom that relates to survival; it shows how addicted people have trouble meeting their needs, which can be assessed by using the following questions.

1 Do they know what they need? (i.e., do their affective states lead them to know what their unmet needs are?).
2 If they know what they need, do they know how to meet their needs?
3 If they know how to meet their needs, are they able to actually meet them?
4 If they are unable to meet their needs, what impedes them to meet them?

These questions indicate that several problems can interfere with a successful satisfaction of internal needs, ranging from the simplest to the most complex ones. Any negative answer to the previous questions could indicate the presence of depression, urging clinicians to explore the patient’s affective functioning. If a conflict remains unsolved for too long, there is certainty that the person is suffering. If parents themselves are unable to work on their own problems, they will show limitations to become models for their children. The use of affect as a guide to look for homeostasis is compromised, thus life itself is compromised and the longer they live in such a context, the worse their affect feels.

The clinical assessment then recruits the two indicators of latent depression suggested in this paper, namely, the depressive affect and the self-aggressive behaviors. The first one should consider the use of manic defenses as an attempt not to experience depressive feelings. When the morbid phase of addiction has begun, it also recruits the chemical manipulation of the original depressive features, which are then harder to assess. Self-aggressive behaviors on the other hand are explicit but not always linked to depressive feelings. Psychoanalytic theories of depression have contributed to emphasize the importance of understanding why people consciously agree to hurt themselves, in spite of being aware of the potential consequences. Some hypotheses have been presented to attempt to understand and treat them, including the use of psychotoxic drugs.

Treatments for addiction benefit from a multidisciplinary approach performed by professional teams that can take into account the various dimensions involved in addiction, i.e., the behavioral, the neurobiological and the subjective. An efficient treatment usually requires several stages. It is useful to acknowledge that addictions in general terms cannot be cured. The brain modifications implied tend to be long-lasting and the rebalancing of the various systems involved has shown limitations. This fact constitutes the most important factor to try to identify the precondition that characterizes the pre-addictive phase and the chronic latent depression that can easily escape clinical attention.

When the addictive phase is active, it is important to use all the available strategies to help addicted patients. Treatment usually entails detoxification, medication, individual and group therapy, abstinence management, self-help groups and behavioral interventions, amongst others. Once the patient is not using drugs, a psychoanalytic psychotherapy can contribute to the patient’s treatment. It requires technical modifications that take into account the complexity implied in addictive disorders. A neuropsychoanalytic approach is highly recommended as seen in treatments reported by Johnson (2009, 2010, 2011), that take into account that addiction is an acquired brain disease (Volkow et al., 2016) and clinicians find themselves in the context of a special type of neurological patient. Not only should the stages of addiction be understood, but also their associated brain damage. Classic psychoanalytic approaches tend to interpret meanings of addiction. As much as those meanings may play a role in the premorbid phase, once the addictive disorder is diagnosed, the brain modifications will play a part that demands more precise interventions. Some modifications can be found in Knight’s proposal to use psychoanalytic techniques to identify the causes of addiction to try to help the patient to find better adaptation strategies (Knight, 1937). Zinberg (1975), in contrast, clarified that the past may be confusing for the patient; he also proposed not to interpret defenses. These two examples represent the various efforts made by several clinicians to help addicted patients. We now know that isolated strategies are not recommended. Team work benefits from the diverse perspectives from which addiction can be treated.

The hypothesis of a latent depression as a precondition of addiction would entail to treat the depressive features, either as prevention against addictive behaviors or as part of the active phase of addiction. If the latent depression remains untreated, the risk of relapse is enhanced. But treating depression is not an easy task either. It feels bad (Zellner et al., 2011) as an evolutionary mechanism that tries to solve the separation distress that is felt as PANIC. This requires mourning for needs that cannot be solved in an ideal way. It has been suggested that addicted patients want to skip the work needed to get satisfying objects and just experience gratification (Solms, 2019). This statement can apply to most cases, but it could also imply that these individuals do not know what to do to achieve what they need. This is particularly important when latent depressions imply a paradox. To accept feelings allows for recognition of the origins of a conflict or need. If a person accepts what cannot be achieved, they may be in a better position to come up with new strategies along with the help of a therapist.

As with any other disorders, the earlier a patient can be helped, the better the outcome. A psychodynamic assessment could pose an opportunity to identify latent depressions, despite the use of numerous defenses, because it is based on the analysis of subjective experience. The transference-countertransference relationship allows for a unique setting to explore the affective configuration of patients. Furthermore, a neuropsychoanalytic approach helps to understand the potential neurobiological implications of a latent depression. This aspect needs further research, but we now know that a neurobiological depressive cascade is linked to subjective feelings that explain behaviors. The consequential addictive cascade yields complex and confusing feelings for patients that go through the different stages of addiction. Depression and addiction are thus, deadly disorders.

In conclusion, the contribution of this paper to the existing treatment models is to emphasize the importance of the subjective feelings of addicted patients. They express an anaclitic conflict that does not find successful solutions. Thus, patients need to tolerate and accept their feelings in order to survive. If they cannot solve their separation distress conflicts in substitutive ways, the negative feelings will not stop. This context constitutes a type of traumatic memory that forms rigid defensive patterns in an attempt to suffer less. New templates should be built with psychotherapeutic help. Yet, they will not delete the old and confusing memories of ambivalent separation distress, but they can help to find better ways to update prediction models and to improve feelings in a legitimate way, i.e., finding alternative actual solutions. Many affective conditionings cannot be modified, still patients can learn to work for what they can have and mourn for what they cannot.

I am aware that these brief suggestions are also insufficient. Clinical observations of patients constitute a valuable opportunity to improve our understanding of the subjective complexity of addiction. This type of observation requires an integrative perspective. The contributions of all disciplines, interested in comprehending the many unsolved features of addiction, should be taken into account to enrich the existing therapeutic approaches. The solution to the depressive paradox entails complicated questions that a psychoanalytically oriented treatment may help to improve in a long-term basis. However, in the case of addicted patients whose brain has become deadly ill, the solution to the addictive paradox is a priority. New predictive patterns should be able to be updated in order to find realistic and legitimate solutions to problems. Their effectiveness should help to inhibit and substitute the original dysfunctional ones. The treatment should teach patients to use feelings as a guide to know what to SEEK. They should work for what they can achieve and mourn what did not and will not happen (Flores Mosri, 2017b). Yet, if people were able to mourn and elaborate loss, depression would present less frequently. The depressive paradox identified by psychoanalytic observations may constitute a valuable opportunity to prevent addiction. But, since it is a paradox, its solution still seems unsolved. Thus, any serious attempt at treating addiction represents hope to keep SEEKING and is most needed.

## Author Contributions

The author confirms being the sole contributor of this work and has approved it for publication.

## Conflict of Interest

The author declares that the research was conducted in the absence of any commercial or financial relationships that could be construed as a potential conflict of interest.

## Abbreviations

ACC: anterior cingulate cortex
BNST: bed nucleus of the stria terminalis
CRF: corticotropin-releasing factor
EOS: endogenous opioid system
HPA: hypothalamic-pituitary-adrenal axis
KOR: kappa opioid receptor
LTD: long term depression
LTP: long term potentiation
NAcc: nucleus accumbens
PAG: periaqueductal gray
PFC: prefrontal cortex
VTA: ventral tegmental area.
